# Hydrophobic Deep Eutectic Solvent‐Enhanced Filaments: A Green Breakthrough for Additive‐Manufactured Electrodes

**DOI:** 10.1002/cssc.202502401

**Published:** 2026-03-22

**Authors:** Karen Kenlderi de Lima Augusto, Elena Bernalte, Robert D. Crapnell, Paulo C. Gomes‐Junior, Bruno Ferreira, Thiago Regis Longo Cesar Paixão, Orlando Fatibello‐Filho, Craig E. Banks

**Affiliations:** ^1^ Faculty of Science and Engineering Manchester Metropolitan University Manchester Great Britain; ^2^ Departamento de Química Universidade Federal de São Carlos São Paulo Brazil; ^3^ Electrochemistry and Fuel Cell Laboratory Federal University of Pará Belém Brazil; ^4^ Departamento de Química Fundamental Instituto de Química Universidade de São Paulo São Paulo Brazil

**Keywords:** acetaminophen, additive manufacturing, cellulose, hydrophobic deep eutectic solvent, sustainability, 3D printing

## Abstract

Advancing sustainable materials for functional devices is a key challenge in green chemistry. This study reports, for the first time, the integration of a hydrophobic deep eutectic solvent (HDES) into a conductive filament composed of recycled polylactic acid (rPLA), carbon black, cellulose, and bio‐based castor oil, yielding a sustainable material with enhanced performance. The resulting composite not only incorporates renewable and recycled components but also exhibits enhanced mechanical and electrochemical performance. Cellulose enhanced the mechanical properties and printability of the filament, while HDES significantly improved its electrical conductivity, reducing bulk resistance from (1.16 ± 0.1) to (0.86 ± 0.02) kΩ representing an improvement over the previously reported CB/cellulose/rPLA electrode, and increased electrochemical performance. Electrochemical characterization of the fabricated electrodes demonstrated superior redox behavior, with enhanced charge‐transfer rates and electroactive surface areas compared to other bespoke filaments. The CB/cellulose/HDES/rPLA electrodes were successfully applied to the voltammetric determination of acetaminophen in water samples, achieving a linear concentration range of 5.0–300 µM and a limit of detection of 0.12 µM. These findings highlight the potential of HDES‐modified filaments in the development of novel, sustainable, high‐performance additive‐manufactured electrochemical sensors, combining additive manufacturing with green materials with enhanced functionality.

## Graphical abstract

This study presents the first use of hydrophobic deep eutectic solvents (HDESs) in a sustainable filament for additive manufacturing electrochemistry. The HDES–cellulose synergy enhances printability and conductivity. Made from recycled and bio‐based materials, the filament offers improved performance with reduced plastic and carbon content, advancing green analytical technologies.

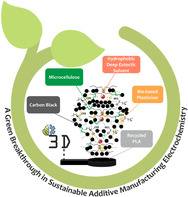

## 1 Introduction

Additive manufacturing, or 3D printing, has emerged as a versatile approach for producing complex geometries that are difficult to achieve using conventional subtractive techniques [1]. Amongst the various additive manufacturing methods, fused filament fabrication (FFF) has become one of the most widely used owing to its relative simplicity, cost‐effectiveness, rapid prototyping, and the fabrication of components with tailored properties, which has attracted growing interest in both academic and industrial settings [2, 3]. The adoption of FFF in the fabrication of electrochemical devices has been particularly promising. The ability to produce electrodes with specific shapes while reducing material waste and minimizing post‐processing aligns well with the growing demand for sustainable manufacturing practices [2, 4].

In line with sustainable manufacturing principles, several studies have explored the use of environmentally responsible materials in order to incorporate practices aligned with the United Nations Sustainable Development Goals, particularly Goal 12, which focuses on sustainable consumption and production [5]. Looking to enhance sustainability, researchers have reported the use of recycled materials and polymers for the fabrication of filaments employed in the development of additive‐manufactured electrodes (AMEs) [6, 7, 8], as well as the incorporation of bio‐based plasticizers [9, 10, 11]. In addition, biomass‐derived fillers such as biochar have been explored as sustainable additives [12, 13]. Furthermore, in efforts to significantly improve the electrochemical performance of AMEs, filaments containing metallic nanoparticles synthesized through eco‐friendly methods have been prepared [14, 15, 16], alongside the addition of naturally derived components such as graphite and cellulose, which contributed not only to enhance the electrochemical properties but also to reduce the overall cost of the produced filaments [17, 18, 19].

In this context, the use of deep eutectic solvents (DESs) is a promising strategy to enhance the electrochemical performance of AMEs while also contributing to the sustainability of newly developed filaments. DESs are well recognized in the literature as green solvents owing to the nature of their constituents and their favorable environmental profile [20, 21]. Most DESs are prepared from readily available, low‐cost, and often biodegradable components such as choline chloride, organic acids, amino acids, or sugars [22]. Their synthesis typically involves a simple mixing process at moderate temperatures (usually ≤100°C) without the need for additional purification steps, thereby minimizing energy consumption and avoiding the use of hazardous reagents [23, 24]. Furthermore, DESs generally exhibit negligible vapor pressure and are non‐volatile, which greatly reduces the release of harmful organic compounds into the atmosphere. They are typically non‐flammable, possess low toxicity, and can often be recycled or reused without significant loss of performance [25, 26]. These attributes are fully aligned with the principles of green chemistry, including waste minimization, the use of safer solvents and auxiliary substances (such as solvents, separation agents, and related compounds), and the design of energy‐efficient processes [27]. Due to these characteristics, DESs have been extensively explored across a broad range of fields, including chromatography [28, 29], separation and extraction processes [30, 31], as well as electrochemistry, particularly in the design and development of electrochemical sensors [32, 33, 34, 35, 36, 37]. Gomes‐Junior et al. employed a DES based on choline chloride and urea to synthesize ultrasmall platinum [38] and cerium nanoparticles [39]. In both studies, the authors highlighted the role of the DES as a stabilizing agent, effectively preventing the metallic nanoparticles agglomeration and thereby yielding smaller particles with enhanced electrochemical properties for use in electrochemical sensor fabrication. Moreover, several studies have highlighted the potential of different DESs as media for the controlled growth of films through the electrodeposition of metal particles and polymers [40, 41, 42, 43]. However, only a few reports have explored the direct incorporation of a DES into an electrode as an active modifier, for instance, by integrating these materials directly into carbon paste electrodes or glassy carbon electrodes [34, 35, 36, 37, 44]. Overall, studies that employ a DES directly as a modifier emphasize its interaction with the conductive material in the electrode, which appears to produce a synergistic effect. This interaction not only enhances the connectivity between the carbonaceous material but can also induce structural modifications in the carbonaceous matrix, such as increasing defect sites and altering surface characteristics, thereby expanding the electroactive area and improving the efficiency of the electrochemical reactions [35, 36, 37].

Building upon these advances, the present work reports, for the first time, the incorporation of a hydrophobic DES (HDES) directly into a thermoplastic filament for the fabrication of AMEs by FFF. By embedding the HDES within the filament formulation, we aim to combine the green attributes and electrochemical benefits of DES with the versatility of additive manufacturing. To validate the approach, the HDES‐containing filaments were used to produce AMEs, which were subsequently applied to the electrochemical determination of acetaminophen in water samples.

## 2 Experimental

### 2.1 Chemicals

All chemicals used in this study were of analytical grade and employed as received, without any further purification. Aqueous solutions were prepared using deionized water with a measured resistivity of not less than 18.2 MΩ cm, obtained from a Milli‐Q Integral 3 system (Millipore UK, Watford, United Kingdom). Hexaamineruthenium(III) chloride (98%), castor oil, potassium ferricyanide (99%), potassium ferrocyanide (98.5%–102%), sodium hydroxide (>98%), potassium chloride (99.0%–100.5%), microcrystalline cellulose (20 µm, 99%), acetaminophen (≥99.0%), tetrabutylammonium bromide (TBAB, ≥99.0%), decanoic acid (DecA, ≥98.0%), and phosphate‐buffered saline (PBS) tablets were purchased from Merck (Gillingham, United Kingdom). Carbon black (>99 + %, <20 nm) was obtained from PI‐KEM (Tamworth, United Kingdom), and recycled poly(lactic acid) (rPLA) was sourced from Gianeco (Turin, Italy). River water samples were collected in accordance with EPA guidelines from the River Irwell in Greater Manchester, United Kingdom (approximate coordinates: 53.517464, −2.302739). Tap water samples were collected from Laboratory 5.39, Dalton Building, Manchester, United Kingdom.

### 2.2 Hydrophobic Eutectic Solvent Preparation

Hydrophobic eutectic mixtures with different molar ratios of the fatty acid (DecA) and TBAB were prepared (Table S1), using a similar procedure reported in the literature [45]. The fatty acid was melted before adding TBAB. The synthesis took place over 5 min in a closed glass vessel while maintaining the temperature at 353.15 K (80°C). The melting point obtained for each eutectic solvent was used to plot a phase diagram, aiming at finding the eutectic point for the studied system.

### 2.3 Thermal Behavior of Eutectic Solvent

Differential scanning calorimetry (DSC) curves were obtained in a Q10 Differential Scanning Calorimetric module, controlled by the Thermal Advantage Series software (v.5.5.24), both from TA Instruments (New Castle, United States). Analyses were carried out using a sample mass of 5.0 ± 0.1 mg, at a heating rate of 10°C min^−1^ and under a dynamic N_2_ atmosphere flowing at 50 mL min^−1^. Closed aluminium sample holders with a pin hole in the centre of the lid (*Ø* = 0.7 mm) were utilized in all experiments. The measurements were performed in the temperature range of 223.15–393.15 K (−50°C–120°C). The endothermic peak temperatures were used to characterize the melting event.

Thermogravimetry/differential thermal analysis curves were obtained in a simultaneous TG/DTA SDT‐Q600 module controlled by the Thermal Advantage software for Q Series v.5.5.24 from TA Instruments (New Castle, United States). The measurements were performed under a dynamic nitrogen atmosphere flowing at 50 mL min^−1^ in temperatures ranging from the ambient to 873.15 K (600°C). After that, the atmosphere was changed to dynamic dry air (flow 50 mL min^−1^) until 1273.15 K (1000°C). A sample mass of (10.0 ± 0.2) mg, a heating rate of 20°C min^−1^, and open *α*‐alumina sample holders were used in all experiments.

### 2.4 Filament Production and Additive Manufacturing of the Electrodes

All commercially acquired recycled PLA was dried in an oven at 60°C for at least 2.5 h to eliminate residual moisture. The polymer composition was prepared by incorporating in a 63 cm^3^ chamber appropriate amounts of rPLA, carbon black (CB), cellulose, castor oil, and HDES. All filaments made throughout this work utilized 10 wt% castor oil as a plasticizer [9]. The proportions of rPLA, CB, and cellulose were optimized based on a previous study [18], with respective contents of 55, 20, and 10 wt%. The HDES content was set at 5 wt%, determined relative to the proportions of the other components. The compounds were mixed using a Thermo Haake Polydrive dynameter fitted with a Thermo Haake Rheomix 600 (Thermo‐Haake, Germany) at 190°C with Banbury rotors at 70 rpm for 5 min. The resulting polymer composites were allowed to cool to room temperature before being granulated to create a finer particle size using a Rapid Granulator 1528 (Rapid, Sweden). The polymer composites were collected and processed through the hopper of an EX2 extrusion line (Filabot, VA, United States). The EX2 was set up with a single screw with two set heat zones of 60°C, and 195°C, respectively. The molten polymer was extruded from a 1.75‐mm die head, pulled along an airpath cooling line (Filabot, VA, United States) and collected on a spool. After this, the filament was ready to use for additive manufacturing.

All computer‐aided designs and corresponding 3MF files presented in this study were created using Fusion 360 (Autodesk, CA, United States). These files were then processed in PrusaSlicer (Prusa Research, Prague, Czech Republic) to generate the G‐code files. The electrodes were fabricated via FFF using a Prusa i3 MK3S+ printer (Prusa Research, Prague, Czech Republic). All electrodes were printed under identical conditions: a 0.6‐mm nozzle, nozzle temperature of 215°C, heat‐bed temperature of 50°C, 100% rectilinear infill [46], 0.15 mm layer height, and a print speed of 35 mm s^−1^.

### 2.5 Physicochemical Characterization and Electrochemical Experiments

Thermogravimetric analysis of the proposed CB/cellulose/HDES/rPLA filament was conducted on a Discovery Series SDT 650 instrument (TA Instruments, DA) operated with Trios software. Samples were placed in alumina pans (90 μL) and subjected to a heating ramp of 10°C min^−1^ from 0°C to 800°C under a nitrogen flow of 100 mL min^−1^.

Scanning electron microscope (SEM) micrographs were obtained using a Crossbeam 350 Focussed Ion Beam–Scanning Electron Microscope (FIB‐SEM) (Carl Zeiss Ltd., Cambridge, United Kingdom) fitted with a field emission electron gun. Secondary electron imaging was completed using a secondary electron secondary ion detector. Samples were mounted on the aluminium SEM pin stubs (12 mm diameter, Agar Scientific, Essex, United Kingdom) using adhesive carbon tabs (12 mm diameter, Agar Scientific, Essex, United Kingdom) and coated with a 5‐nm layer of Au/Pd metal using a Leica EM ACE200 coating system before imaging.

X‐ray photoelectron spectroscopy (XPS) data were acquired using an AXIS Supra (Kratos, United Kingdom), equipped with a monochromatic Al X‐ray source (1486.6 eV) operating at 225 W and a hemispherical sector analyser. It was operated in fixed transmission mode with a pass energy of 160 eV for survey scans and 20 eV for region scans with the collimator operating in slot mode for an analysis area of approximately 700 × 300 μm. The FWHM of the Ag 3d_5/2_ peak using a pass energy of 20 eV was 0.613 eV. The binding energy scale was calibrated by setting the graphitic sp^2^ C 1s peak to 284.5 eV; this calibration is acknowledged to be flawed [47] but was nonetheless used in the absence of reasonable alternatives, because only limited information was to be inferred from absolute peak positions.

All electrochemical experiments were performed on an Autolab 100 N potentiostat controlled by NOVA 2.1.7 (Utrecht, The Netherlands). Identical AMEs were used throughout this work for all filaments, printed in a lollipop shape (*Ø* 5‐mm disk with 8 mm connection length and 2 × 1 mm thickness [48]) alongside an external commercial Ag|AgCl/KCl (3.0 M) reference electrode with a nichrome wire counter electrode. All solutions of [Ru(NH_3_)_6_]^3+^ were purged of O_2_ thoroughly using N_2_ prior to any electrochemical experiments. Solutions of [Fe(CN)_6_]^4−/3−^ were prepared in the same way without the need of further degassing. Electrochemical impedance spectroscopy (EIS) was recorded in the frequency range 0.1 Hz to 100 kHz applying 10 mV of signal amplitude to perturb the system under quiescent conditions. NOVA 2.1.7 software was used to fit the Nyquist plots to an adequate equivalent circuit.

Activation of the AMEs was performed before electrochemical experiments. This was achieved electrochemically in NaOH (0.5 M), as described in the literature [49]. Briefly, the AMEs were connected as the working electrode in conjunction with a nichrome wire coil counter and Ag|AgCl/KCl (3.0 M) reference electrode and placed in a solution of 0.5 M NaOH. Chronoamperometry was used to activate the AMEs by applying a set voltage of +1.4 V for 200 s, followed by applying −1.0 V for 200 s. The AMEs were then thoroughly rinsed with deionized water and dried under compressed air before further use.

Square‐wave voltammetry (SWV) was used to the analytical determination of acetaminophen under the following conditions: frequency (*f*) = 25 Hz, step potential (Δ*E*) = 5.0 mV, and amplitude potential (*a*) = 20 mV.

## 3 Results and Discussion

### 3.1 Thermal Analysis and Phase Behavior of the Hydrophobic Eutectic Solvent

DSC analysis was employed to determine the melting temperatures of various DecA/TBAB‐based mixtures (Table S1) for the construction of a phase diagram and identification of the eutectic point. The DSC curves for all mixtures are shown in Figures S1A–J and 1A. The thermal events of the pure components (DecA and TBAB) were reported in our previous work [35]. Key thermal events were identified, including melting temperature (*T* *_m_*), phase transition temperature (*T*_trans_), and the temperature associated with the free fraction of the individual components (DecA or TBAB), denoted as T*.

**FIGURE 1:**
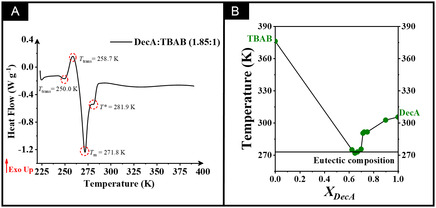
(A) DSC curve with the thermal events for the HDES at the eutectic point (1.85:1). *T*_trans_: temperature of phase transition; *T* *_m_*: melting temperature; T*: temperature of the free fraction of DecA. (B) Phase diagram of the DecA/TBAB eutectic system as a function of the fatty acid molar ratio.

The DSC curve for HDES at the eutectic composition (Figure 1A) exhibits four thermal events during heating. Two initial events at 250.0 K (−23.15°C, endothermic) and 258.7 K (−14.45°C, exothermic) correspond to a solid–solid phase transition, commonly associated with the transformation from an amorphous to a crystalline solid in fatty acid‐based eutectic systems [50, 51]. This transition was also observed for the other fractions of the mixtures studied (Figure S1). The third thermal event, at 271.8 K (−1.35°C), can be attributed to the melting temperature of the crystalline phase, which refers to the eutectic point for the DecA/TBAB system. Regarding the last thermal event, an endothermic peak close to 281.9 K (8.75°C) was observed, and its occurrence may be related to the increase in acid or TBAB fractions that may not have been incorporated into the hydrophobic mixture. Similar findings were reported by Shishov et al. [52], who characterized eutectic mixtures composed of nonanoic acid and tetrabutylammonium bromide. They attributed the appearance of a peak near 277 K (3.85°C) to the presence of unbound (free) nonanoic acid. The 1.50:1 composition for HDES (Figure S1D) showed a broad endothermic event at 265.5 K (−7.65°C). Nevertheless, this event should not be interpreted as the eutectic point, as similar phase transitions associated with fatty acid crystallization were consistently observed at temperatures close to 260 K (−13.15°C).

The melting behavior of the DecA/TBAB system was represented in a phase diagram as a function of the DecA molar ratio (Figure 1B), showing a characteristic melting point depression and a V‐shaped profile typical of eutectic systems [52, 53].

The thermal behavior of the HDES at the eutectic composition (1.85:1) and its precursors was evaluated by thermogravimetric analysis under a nitrogen atmosphere (Figure S2). The distinct thermal profiles of the HDES compared to its individual components indicate interactions between the precursors at the eutectic point. Melting events at 376 K (103°C) for TBAB and 305 K (32°C) for DecA, consistent with DSC data, were absent in the HDES, which melts below room temperature. DecA showed 98.6% mass loss at 492 K (219°C) and TBAB 98.8% at 499 K (226°C), with an additional minor event near 540 K (267°C) likely related to TBAB impurities. For the HDES at the eutectic point, decomposition occurred at 506 K (233°C) with 99.4% mass loss, indicating improved thermal stability compared to its individual components.

### 3.2 Preparation and Characterization of the HDES‐Based Additive‐Manufactured Electrodes

The application of DESs to sensors development is growing considerably due to their favorable physicochemical properties and their capacity to enhance electrode performance [32, 33, 34, 35, 37, 54, 55]. By promoting interactions between the conductive materials, DES can improve conductivity and enhance sensitivity for analyte detection [34, 35, 36, 37]. In this work, the synthesized HDES composed of decanoic acid and tetrabutylammonium bromide was selected for novel filament preparation. It is important to mention that the low water content of this HDES makes it compatible to mix with PLA [56, 57], while also contributing to improve sustainability and electrochemical properties, as supported by the literature [32, 35, 58]. The preparation of the HDES‐modified filament was based on a previously reported method [18]. Here, the HDES was combined with carbon black, castor oil, recycled PLA, and cellulose to produce a novel HDES‐based filament, named as CB/cellulose/HDES/rPLA. The formulation consisted of 20 wt% CB, 10 wt% castor oil, 5 wt% HDES, 10 wt% cellulose, and 55 wt% rPLA.

It was observed that the incorporation of cellulose in this new sustainable filament was critical to maintain the structural integrity of the CB/cellulose/HDES/rPLA filament that could not be achieved otherwise. The eutectic solvent used is acidic, which can compromise the mechanical properties of PLA [59, 60] by increasing brittleness and reducing flexibility, hindering the printing process. An initial formulation attempt using only HDES, castor oil, rPLA, and CB (CB/HDES/rPLA) resulted in a filament with insufficient flexibility for 3D printing. To overcome this limitation, cellulose was added to the mixture, as it is well known that cellulose improves the mechanical performance of PLA, acting as a reinforcing agent and enhancing the overall printability and durability of the filament [61]. The enhanced flexibility achieved for the CB/cellulose/HDES/rPLA filament, as well as its superior printability compared to the CB/HDES/rPLA formulation, is illustrated in Figure S3. Cellulose proved to be a key component during the printing process. Its incorporation enabled the consistent fabrication of small, well‐defined disk‐shaped electrodes, as the CB/cellulose/HDES/rPLA filament could withstand the rapid printer movements required for generating such geometries, demonstrating that cellulose contributes not only to improved material properties but also to reliable printability. In addition to these advantages, the CB/cellulose/HDES/rPLA filament exhibited enhanced electrical conductivity. Over a 10‐cm length, the filament displayed a low average bulk resistance of (0.86 ± 0.02) kΩ, representing a significant improvement compared to commercially available conductive PLA, which typically exhibits resistance values between 2 and 3.5 kΩ [62]. This value is also lower than that previously reported for a cellulose‐containing filament, which showed a resistance of (1.16 ± 0.10) kΩ [18], as well as for the CB/HDES/rPLA filament, (1.36 ± 0.10) kΩ, confirming the beneficial synergy between cellulose and HDES.

**FIGURE 2:**
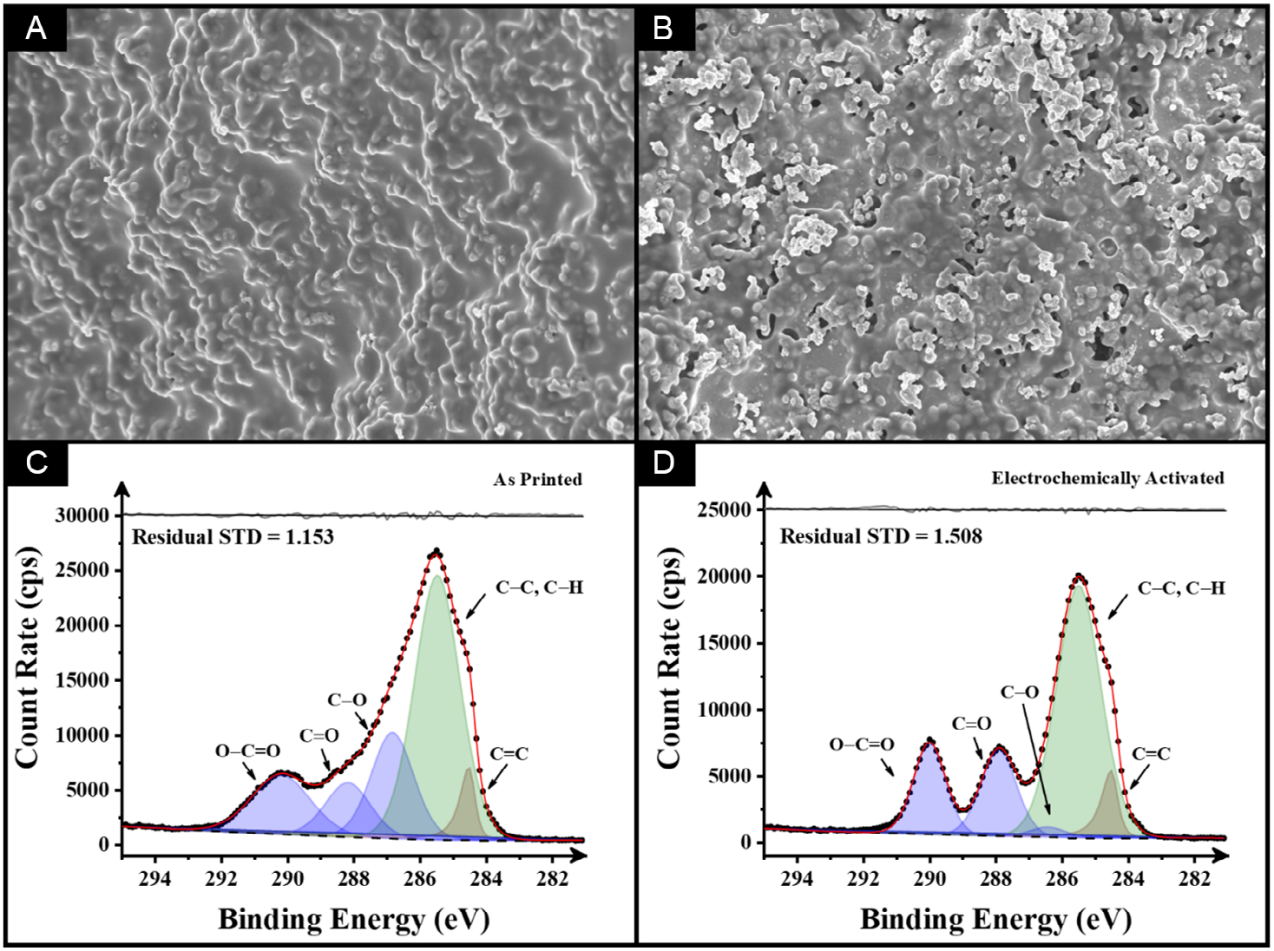
SEM surface images and XPS for the (A,C) as‐printed and (B,D) activated CB/cellulose/HDES/rPLA electrode.

After 3D‐printing the electrodes, it is essential to characterize the surface of them to better understand their performance. When employing PLA‐based materials, surface activation is a common practice. This process involves removing the outer polymer layer that coats the electrode surface, thereby exposing a greater amount of the conductive filler. Electrochemical activation in a 0.5‐M sodium hydroxide solution is amongst the most widely used methods and was employed throughout this study [49]. Subsequently, the AMEs were analyzed using SEM and XPS before (as‐printed) and after electrochemical activation, as shown in Figure 2.

The effectiveness of the activation process is evident in the surface morphology of the electrodes. As shown in the SEM images (Figure 2A,B), the presence of filler morphology is evident, but it is covered by a smooth polymer layer due to the gloving effect during material extrusion. After activation, distinct and well‐defined filler components can be observed, indicating the removal of this superficial polymeric layer.

Furthermore, the chemical composition of the CB/cellulose/HDES/rPLA electrode was analyzed using XPS. Figure 2C,D displays the C 1s spectra for as‐printed and electrochemically activated electrodes in 0.5 M NaOH, respectively. To achieve an accurate spectral fit, five peaks were assigned. The primary asymmetric peak at 284.5 eV corresponds to the X‐ray photoemission of graphitic carbon [63, 64]. Additionally, four symmetric peaks were required to fit the data, representing *sp* ^3^ C—C/C—H, C—O, C=O, and O—C=O bonding. As shown in Figure 2C,D, the dominant intensity of the C—C/C—H peak is expected due to the inherent structure of rPLA and castor oil. In rPLA, the C—C, C—O, and O—C=O groups are present in comparable proportions, resulting in C 1s peaks of similar intensity. In contrast, castor oil contains a much higher proportion of aliphatic C—C/C—H bonds relative to C—O and O—C=O, which explains the increased intensity of this component in the spectrum [9]. The additional contributions from the C—O, C=O, and O—C=O peaks observed in the as‐printed CB/cellulose/HDES/rPLA electrode can be attributed to the chemical nature of both the cellulose and the HDES used in the formulation. Cellulose is rich in hydroxyl (—OH) groups [18], contributing significantly to C—O bonding, while the HDES, composed of tetrabutylammonium bromide and decanoic acid, also contains oxygenated moieties [45]. The C 1s spectral fitting indicates that, in the as‐printed electrode, the C—C/C—H peak at 285.0 eV accounts for approximately 50% of the atomic concentration, compared to 20%, 9%, and 14% for the C—O, C=O, and O—C=O peaks, respectively (Table 1). Upon electrochemical activation, the XPS C 1s spectrum exhibited an increased intensity of the graphitic C=C peak, suggesting greater exposure of conductive materials. Additionally, the intensities of the C=O and O—C=O bonds were enhanced, further supporting the improved accessibility of electroactive sites influenced by the HDES, as the decanoic acid component contains these groups.

**TABLE 1:** The atomic concentration of the species in each electrode.

CB/cellulose/HDES/rPLA	Atomic concentration, %					
	C=C	C—C/C—H		C—O	C=O	O—C=O
As‐printed	5.7	49.8		20.6	9.12	14.8
Activated	7.3	59.4		1.9	16.1	15.4

Furthermore, the thermogravimetric analysis of the bespoke filament was performed and is shown in Figure S4. The HDES‐based filament exhibited an onset temperature of thermal degradation at 270.8°C, indicating a slight decrease compared to the onset temperature reported previously for a CB/cellulose/PLA‐based filament (279°C) [18]. This suggests that the addition of the acidic HDES somewhat compromised the thermal stability of the filament. The degradation process was maximum at 352.2°C, with a total mass loss of 68.7%. The filler content of the filament was calculated to be (26 ± 2) wt%; considering a filler fraction of 35 wt% (CB + cellulose + HDES), the lower value was expected, as the HDES undergoes substantial mass loss below 352°C. These results are consistent with the 20 wt% CB content in the filament, and the remaining filler fraction can be attributed to residual cellulose in the system [18].

### 3.3 Electrochemical Characterization of the Additive‐Manufactured Electrodes

**FIGURE 3:**
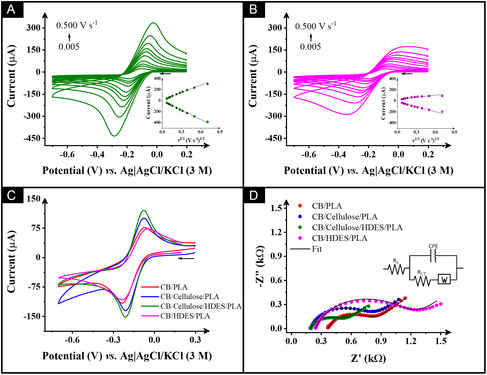
Scan rate study (5–500 mV s^−1^) with 1.0 mM [Ru(NH_3_)_6_]^3+^ in 0.1 M KCl performed using (A) CB/cellulose/HDES/rPLA and (B) CB/HDES/rPLA electrodes as the WE. Inset: The Randles–Ševčík plot. (C) Cyclic voltammograms (50 mV s^−1^) in [Ru(NH_3_)_6_]^3+^ (1 mM in 0.1 M KCl) and (D) EIS Nyquist plots in [Fe(CN)_6_]^4−/3−^ (1 mM in 0.1 M KCl) performed with (—) CB/PLA, (—) CB/cellulose/rPLA, (—) CB/cellulose/HDES/rPLA, and (—) CB/HDES/rPLA.

The CB/cellulose/HDES/rPLA and CB/HDES/rPLA electrodes were characterized using the near‐ideal outer‐sphere redox probe hexaamineruthenium(III) chloride, [Ru(NH_3_)_6_]^3+^. Representative scan‐rate studies (5–500 mV  s^−1^) for the CB/cellulose/HDES/rPLA and CB/HDES/PLA electrodes are shown in Figure 3A,B, respectively. The results reveal that the combined incorporation of HDES and cellulose into the filament leads to a sharper and significantly better‐defined redox response compared to the CB/HDES/rPLA 3D‐printed electrode. This improvement is evident when comparing the voltammograms recorded at 50 mV  s^−1^ for both electrodes and those of other bespoke electrodes (CB/PLA and CB/cellulose/rPLA). As shown in Figure 3C and Table 2, the CB/cellulose/HDES/rPLA electrode exhibits superior electrochemical parameters relative to all other electrodes evaluated. Specifically, the heterogeneous electron charge‐transfer rate constant (*k* ^0^) and electrochemical surface area (*A*_e_) values for the CB/cellulose/HDES/rPLA electrode were (1.62 ± 0.09) × 10^−3^ cm s^−1^ and (0.72 ± 0.03) cm^2^, respectively, whereas the previously reported CB/cellulose/PLA electrode exhibited a *k* ^0^ of (1.55 ± 0.05) × 10^−^^3^ cm s^−1^ and an *A*_e_ of (0.65 ± 0.09) cm^2^ [18].

**TABLE 2:** Comparison of the cathodic peak current (−*I*_cp_), electrochemically active area (*A*_e_), heterogeneous electron transfer (*k* ^0^), peak‐to‐peak separations (Δ*E*_p_), charge‐transfer resistance (*R*_ct_), and solution resistance (*R*_s_) for the bespoke electrodes.

Electrode	−*I*_cp_, µA^a^	*A*_e_, cm^2^	*k* ^0^, cm s^–1^	Δ*E*_p_, V^a^	*R*s, kΩ		*R*_ct_, kΩ
CB/PLA	109 ± 9	0.59 ± 0.03	(1.31 ± 0.13) × 10^−3^	0.147 ± 0.011	0.33 ± 0.05		0.58 ± 0.02
CB/cellulose/rPLA	116 ± 16	0.65 ± 0.09	(1.55 ± 0.05) × 10^−3^	0.130 ± 0.001	0.19 ± 0.02		0.61 ± 0.10
CB/HDES/rPLA	89 ± 5	0.48 ± 0.03	(1.02 ± 0.08) × 10^−3^	0.175 ± 0.008	0.24 ± 0.01		0.41 ± 0.08
CB/cellulose/HDES/rPLA	133 ± 5	0.72 ± 0.03	(1.62 ± 0.09) × 10^−3^	0.128 ± 0.004	0.20 ± 0.02		0.92 ± 0.02

a 50 mV s^−1^.

As it could be anticipated, modifying the filament with HDES without the addition of cellulose (CB/HDES/PLA) had a negative effect on the PLA matrix and consequently reduced the electrochemical performance due to the acidic nature of the HDES. This electrode exhibited inferior performance compared with the other electrodes, thereby confirming the beneficial role of cellulose in enhancing the filament characteristics.

EIS was employed to further elucidate the interfacial properties of the 3D‐printed electrodes. The Nyquist plots obtained using [Fe(CN)_6_]^4−/3−^ (1 mM in 0.1 M KCl) for the bespoke electrodes are present in Figure 3D. The Nyquist plots were fitted using an equivalent circuit comprising the solution resistance (*R*_s_), the charge‐transfer resistance (*R*_ct_), a constant phase element (CPE) representing the double‐layer behavior, and a Warburg element (*W*) included to represent diffusion limitations within the system. As summarized in Table 2, the bespoke CB/cellulose/HDES/rPLA electrode exhibited the lowest *R*_ct_, confirming an enhanced electron‐transfer kinetics at the electrode–electrolyte interface. This significant reduction in *R*_ct_ is attributed to the synergistic combination of HDES and cellulose, which likely improves the dispersion of CB within the PLA matrix and promotes a more conductive network. In contrast, the CB/HDES/PLA electrode presented a markedly higher *R*_ct_, indicating hindered charge transfer. This behavior is consistent with the results related to the effect of acidic HDES on the structural integrity of PLA.

**SCHEME 1:**
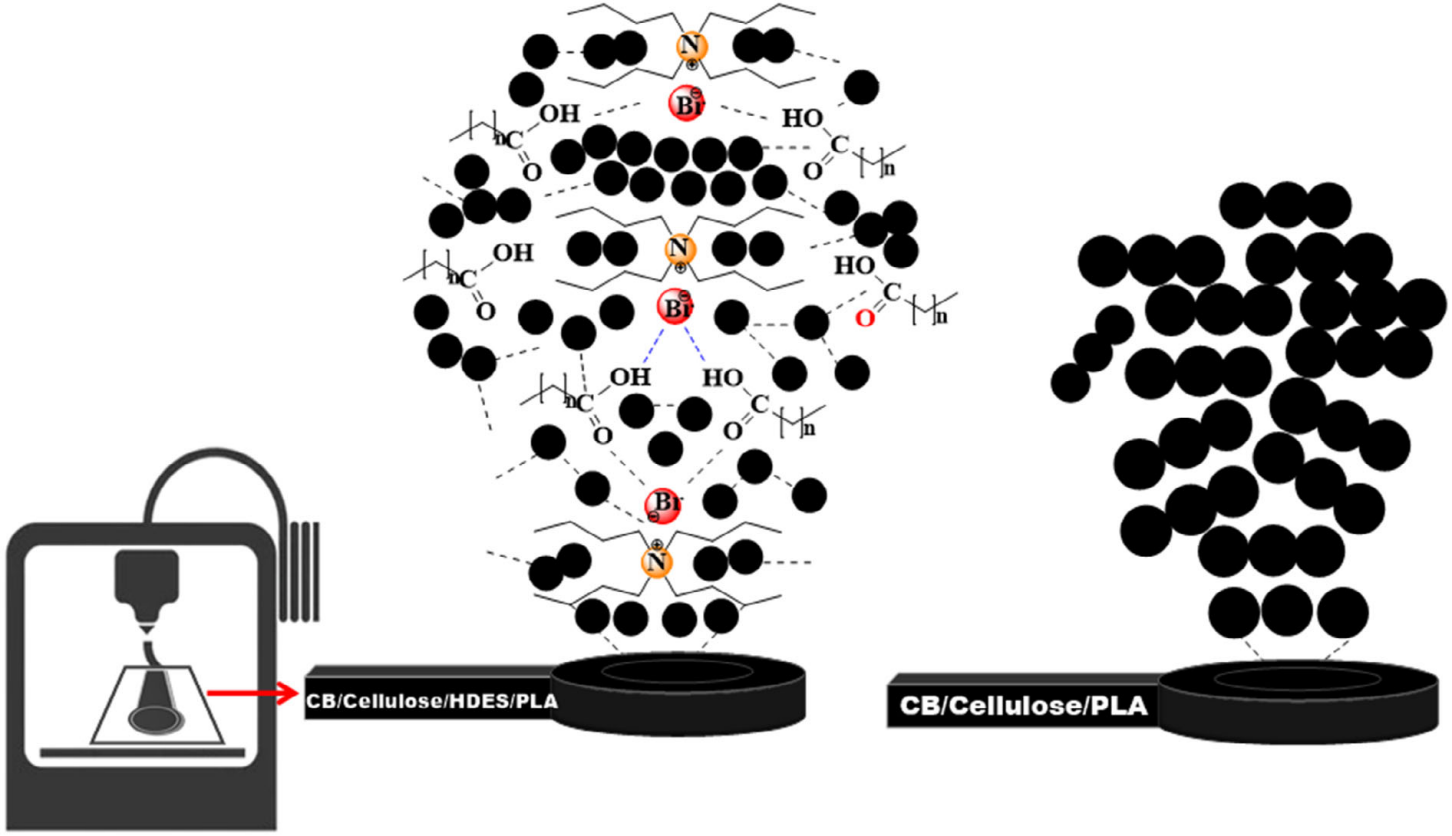
Representation of the presence of HDES in the CB/cellulose/HDES/rPLA electrode, where it acts as a modifier of the nanocarbon particles compared with the CB/cellulose/rPLA electrode.

The improved electrochemical performance is illustrated in Scheme 1 by comparing the CB/cellulose/HDES/rPLA and CB/cellulose/rPLA electrodes. The presence of HDES in the composite strengthens the interconnection amongst the carbonaceous particles. Previous study [35], using atomic force microscopy and electrochemical analyses, showed that DESs can form a uniform, thin coating on carbon nanoparticles, hence enhancing particle–particle contact and facilitating electron transfer. In addition, eutectic solvents can act as surface modifiers, introducing functional groups that accelerate electron‐transfer kinetics, create structural defects, and increase the overall reactivity of the material [65, 66, 67].

### 3.4 Electrochemical Determination of Acetaminophen

**FIGURE 4:**
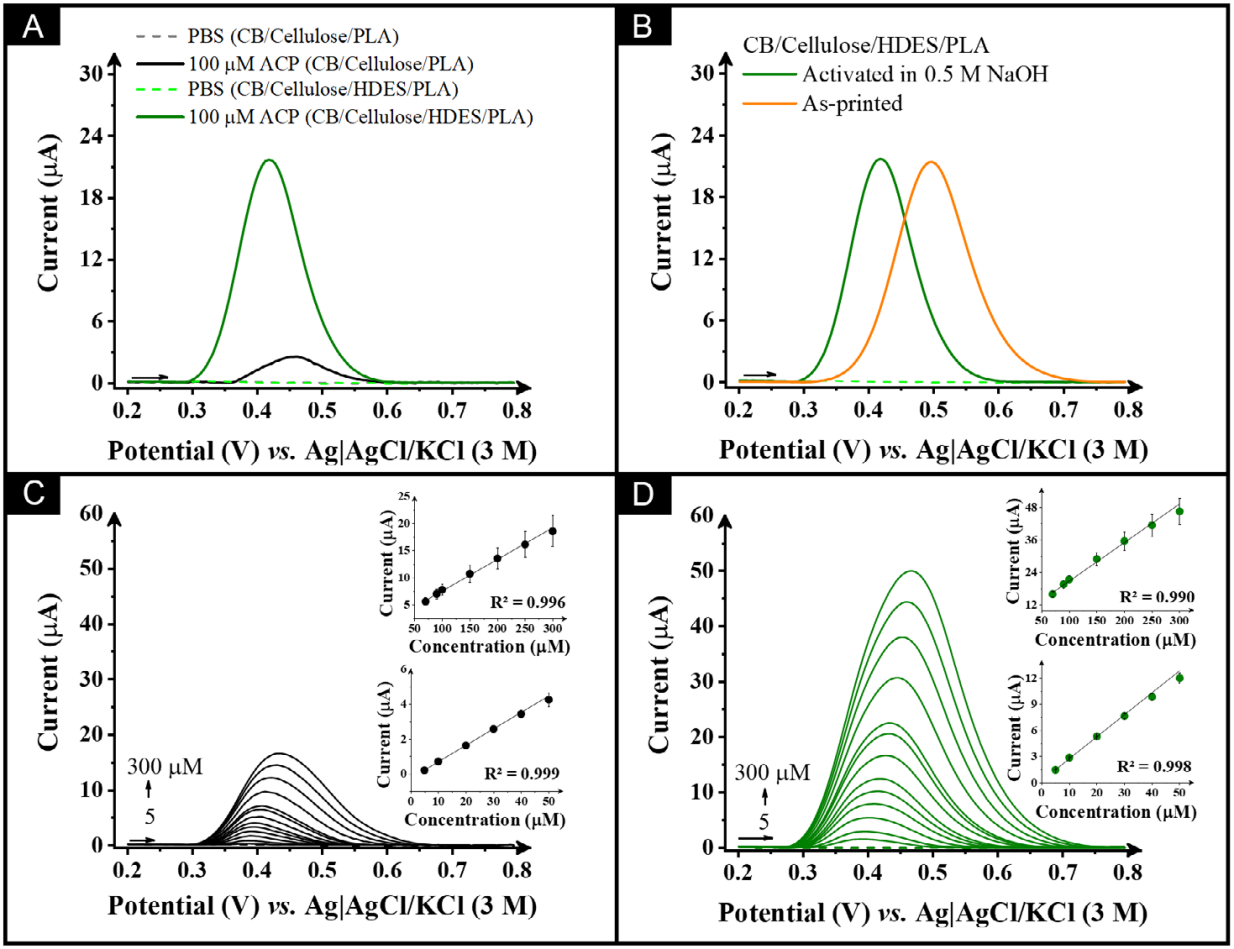
SW voltammograms with 100 µM acetaminophen (ACP) in PBS pH 7.55 (A) comparing the CB/Cellulose/PLA and CB/cellulose/HDES/rPLA electrodes as the WE and (B) comparison between as‐printed and NaOH‐activated CB/cellulose/HDES/rPLA electrodes. SW voltammograms for ACP determination using (C) CB/cellulose/PLA and (D) CB/cellulose/HDES/rPLA electrodes in PBS (pH 7.55), with concentrations ranging from 5.0 to 300 µM. Inset: the calibration plots. SWV parameters: *a* = 20 mV, *f* = 25 Hz, Δ*E* = 5 mV.

After electrochemical characterization, the CB/cellulose/HDES/rPLA composite was employed for the electroanalytical detection of acetaminophen (ACP), a widely used over‐the‐counter analgesic whose frequent and often unregulated consumption has raised notable environmental concerns. In this context, developing sensitive and reliable sensors to monitor ACP levels in water is essential for assessing its environmental impact and ensuring water quality [68]. The results obtained using this electrode were also compared to those from CB/cellulose/rPLA to better illustrate the improvements due to the presence of the HDES. Prior to the electrochemical experiments, the electrodes were activated electrochemically in 0.5 M NaOH [49]. The electrochemical behavior of ACP on the CB/cellulose/HDES/rPLA and CB/cellulose/rPLA electrodes was evaluated using SWV. Figure 4A presents the voltammetric profile of ACP on these electrodes. The CB/cellulose/HDES/rPLA electrode exhibited a significant enhancement in peak current value, with an 8.7‐fold increase in peak current compared to the electrode without HDES. This improvement is likely due to the HDES enhancing the affinity between the analyte and the electrode surface, increasing the electrochemical response. Furthermore, Figure 4B highlights the effect of NaOH activation on the electrochemical response of the electrode. Post‐activation, the oxidation peak potential for ACP shifted from +0.50 to +0.42 V. This shift towards a lower potential suggests improved electron transfer kinetics, which is likely due to the exposure of more conductive regions within the electrode and the enhanced availability of active sites provided by the HDES. These changes are supported by the SEM images, indicating a more accessible surface post‐activation.

To evaluate the electroanalytical performance of the CB/cellulose/HDES/rPLA electrode, acetaminophen detection was conducted and compared to the CB/cellulose/rPLA electrode. The SW voltammograms for acetaminophen detection over the concentration range of 5.0–300 µM are shown in Figure 4C,D, with the corresponding calibration plots showed as an inset. A linear relationship was observed between the peak current and concentration, yielding a theoretical limit of detection of 0.12 and 0.63 µM and a limit of quantification of 0.39 and 2.11 µM for CB/cellulose/HDES/rPLA and CB/cellulose/PLA, respectively. Furthermore, the CB/cellulose/HDES/rPLA electrode exhibited a sensitivity of (0.26 ± 0.01) µA µM^−1^, representing a more than 2.7‐fold increase compared to the CB/cellulose/PLA electrode (0.095 ± 0.001) µA µM^−1^. Additionally, the results of this study were compared to those previously reported in the literature for acetaminophen determination, as summarized in Table S2. The proposed electrode demonstrated excellent analytical performance, even without optimization of the SWV conditions, highlighting its robustness and practical applicability. Furthermore, the fabrication process is simpler and more sustainable, relying on green materials such as DES, cellulose, castor oil, and recycled PLA, eliminating the need for complex modification steps, and offers an environmentally friendly alternative to conventional electrode designs.

Following, a repeatability study was performed on the CB/cellulose/HDES/rPLA electrode, as presented in Figure S5. The results demonstrated a standard deviation of less than 5% for *I*_ap_ across 10 consecutive SWV measurements (Figure S5A). Inter‐electrode reproducibility was also evaluated using three electrodes printed from the same CB/cellulose/HDES/rPLA filament (Figure S5B), yielding consistently low deviations, with a standard deviation of 5.6% for *I*_ap_. These results highlight the reliability and stability of the additive‐manufactured CB/cellulose/HDES/rPLA electrodes for ACP detection.

**FIGURE 5:**
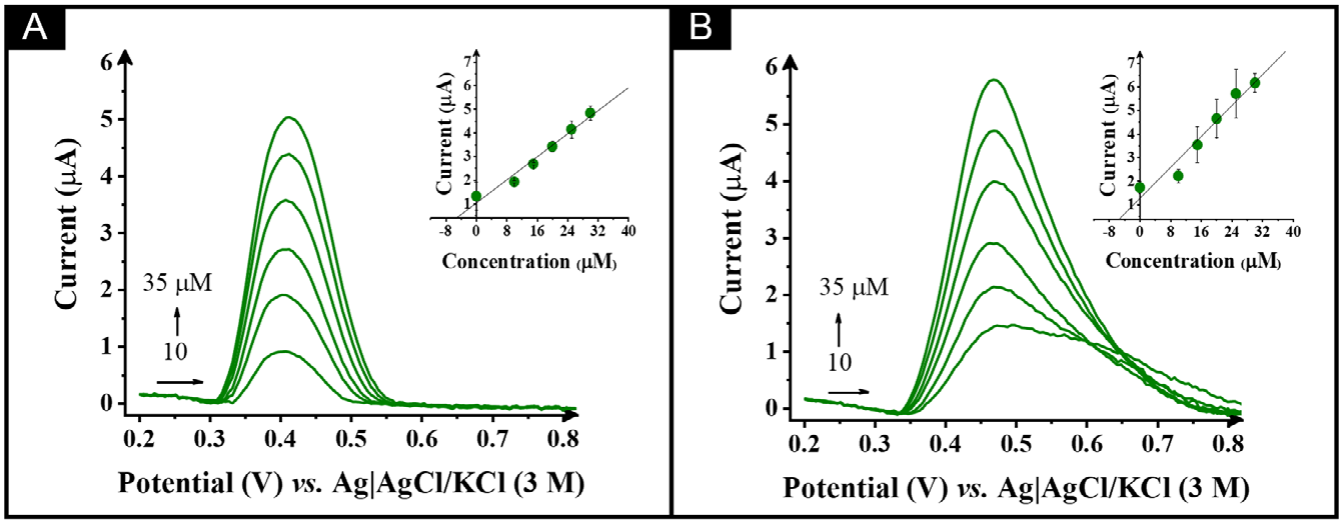
SW voltammograms for ACP detection in spiked (A) diluted river water (50‐fold) and (B) diluted tap water (20‐fold) in PBS pH 7.55 performed at CB/cellulose/HDES/rPLA electrode in a concentration range of 10–35 µM. SWV parameters: *a* = 20 mV, *f* = 25 Hz, and Δ*E* = 5 mV. Inset: calibration curve obtained by the standard addition method.

To finish, the electroanalytical performance of the CB/cellulose/HDES/rPLA electrode was tested in environmental water samples to assess its practical applicability in real‐world scenarios. Figure 5 shows the SWV recorded for the determination of ACP in two different water samples (river water and tap water), which did not undergo any further pre‐treatment. Then, the quantification of ACP in these samples was carried out using the standard addition method. This involved the successive addition of a standard ACP solution at concentrations of 10, 15, 20, 25, 30, and 35 µM to the electrochemical cell. The results confirm the successful detection of ACP in both water samples, with recovery rates of 80% for tap water and 87% for river water.

## 4 Conclusions

This study presents the successful and novel integration of a HDES into the formulation of a conductive filament for 3D printing, marking a significant advancement in sustainable AME fabrication. The resulting CB/cellulose/HDES/rPLA electrode exhibited superior electrochemical performance compared to different combination of bespoke filaments and was effectively applied for the determination of acetaminophen (ACP) in real water samples. These findings emphasize the potential of integrating environmentally friendly chemical strategies within additive manufacturing electrochemistry. By combining ‘green’ materials, such as DES, bio‐based castor oil, recycled PLA, and cellulose, the proposed approach achieves both environmental sustainability and improved electroanalytical functionality of the sensors produced.

Looking forward, this work opens promising avenues for further research that should focus on improving the compatibility between DES and thermoplastic matrices. The observed degradation of PLA due to the acidic nature of the HDES indicates a need to explore alternative DES formulations with improved polymer compatibility or to investigate other thermoplastic materials to optimize the DES–polymer interaction. Addressing these challenges will be key to expanding the applicability of this green approach in next‐generation sensor development.

## Supporting Information

Additional supporting information can be found online in the Supporting Information section. **Supporting Fig. S1**: DSC curves with the thermal events for the different mixtures of DecA/TBAB. *T*_trans_: temperature of phase transition; *T* *_m_*: melting temperature; T*: temperature of the free fraction of the precursors (DecA or TBAB). **Supporting Fig. S2**: TG curves (solid lines) of HDES (DecA/TBAB, 1.85:1), decanoic acid (DecA), and tetrabutylammonium bromide (TBAB). DTG curves (dotted lines) of the HDES and its precursors (TBAB and DecA). Conditions: N_2_ at 50 ml min^−1^, sample mass of (10.0 ± 0.2) mg in open *α*‐alumina sample holders. **Supporting Fig. S3**: Images of the bespoke (A) CB/cellulose/HDES/PLA and (B) CB/HDES/PLA filaments. **Supporting Fig. S4**: TG (black line) and DTG (green line) curves of the HDES‐based filament. Conditions: N_2_ at 100 mL min^−1^. **Supporting Fig. S5**: SW voltammograms obtained for repetitive measurements (*N *= 10) in PBS pH 7.55 with 100 µM acetaminophen using (A) one electrode and (B) three different CB/cellulose/HDES/rPLA electrodes as WE. SWV parameters: *a *= 20 mV, *f *= 25 Hz, and *ΔE *= 5 mV. **Supporting Table S1**: Molar ratio of the obtained hydrophobic mixtures based on decanoic acid and tetrabutylammonium bromide. **Supporting Table S2**: Comparison of the analytical parameters for the voltammetric determination of acetaminophen.

## Author Contributions

**Karen Kenlderi de Lima Augusto**: conceptualization, data curation, formal analysis, investigation, methodology, validation, writing – original draft, writing – review & editing. **Elena Bernalte**: conceptualization, data curation, formal analysis, investigation, methodology, supervision, validation, writing – original draft (equal), writing – review & editing. **Robert D. Crapnell**: conceptualization, formal analysis, investigation, resources, supervision, writing – original draft (equal), writing – review & editing. **Paulo C. Gomes‐Junior**: investigation, methodology, writing – original draft (equal), writing – review & editing. **Bruno Ferreira**: formal analysis, investigation. **Thiago Regis Longo Cesar Paixão**: conceptualization, supervision. **Orlando** **Fatibello‐Filho**: conceptualization, supervision (equal). **Craig E. Banks**: project administration, supervision, writing – review & editing.

## Conflicts of Interest

The authors declare no conflicts of interest.

## Supporting information

Supplementary Material

## Data Availability

The data that support the findings of this study are available from the corresponding author upon reasonable request.
