# Supplementary material for: Hydrophobic Deep Eutectic Solvent‐Enhanced Filaments: A Green Breakthrough for Additive‐Manufactured Electrodes
Source: ChemSusChem. 2026 Mar 22;19(6):e202502401. doi: 10.1002/cssc.202502401 (PMC13006166; doi:10.1002/cssc.202502401)
Supplement: Supplementary file 1 — Supplementary Material [file CSSC-19-e202502401-s001.pdf]

Supporting information

Hydrophobic Deep Eutectic Solvent-Enhanced Filaments: A Green Breakthrough for Additive Manufactured Electrodes

Karen K. L. Augusto[1,2], Elena Bernalte[1], Robert D. Crapnell[1], Paulo C. Gomes-Junior[2], Bruno Ferreira[1,3], Thiago R.L.C. Paixão[3], Orlando Fatibello-Filho[2], and Craig E. Banks[1*]

[1]Faculty of Science and Engineering, Manchester Metropolitan University, Dalton Building, Chester Street, M1 5GD, Great Britain.

[2]Departamento de Química, Universidade Federal de São Carlos, São Carlos, São Paulo, Brazil.

[3]Departamento de Química Fundamental, Instituto de Química, Universidade de São Paulo, São Paulo, Brazil.

[*]To whom correspondence should be addressed.

E-mail address: c.banks@mmu.ac.uk (C.E. Banks) Tel: +44(0)1612471196

Table S1. Molar ratio of the obtained hydrophobic mixtures based on decanoic acid and tetrabutylammonium bromide

Molar Ratio (DecA:TBAB)	Components	
	DecA	TBAB
1:0	1.000	0.000
9:1	0.900	0.100
3:1	0.750	0.250
2.6:1	0.725	0.275
2.5:1	0.715	0.285
2.3:1	0.700	0.300
1.85:1	0.650	0.350
1.67:1	0.625	0.375
1.5:1	0.600	0.400
1.22:1	0.550	0.450
1.1:1	0.525	0.475
1:1	0.500	0.500
0:1	0.000	1.000

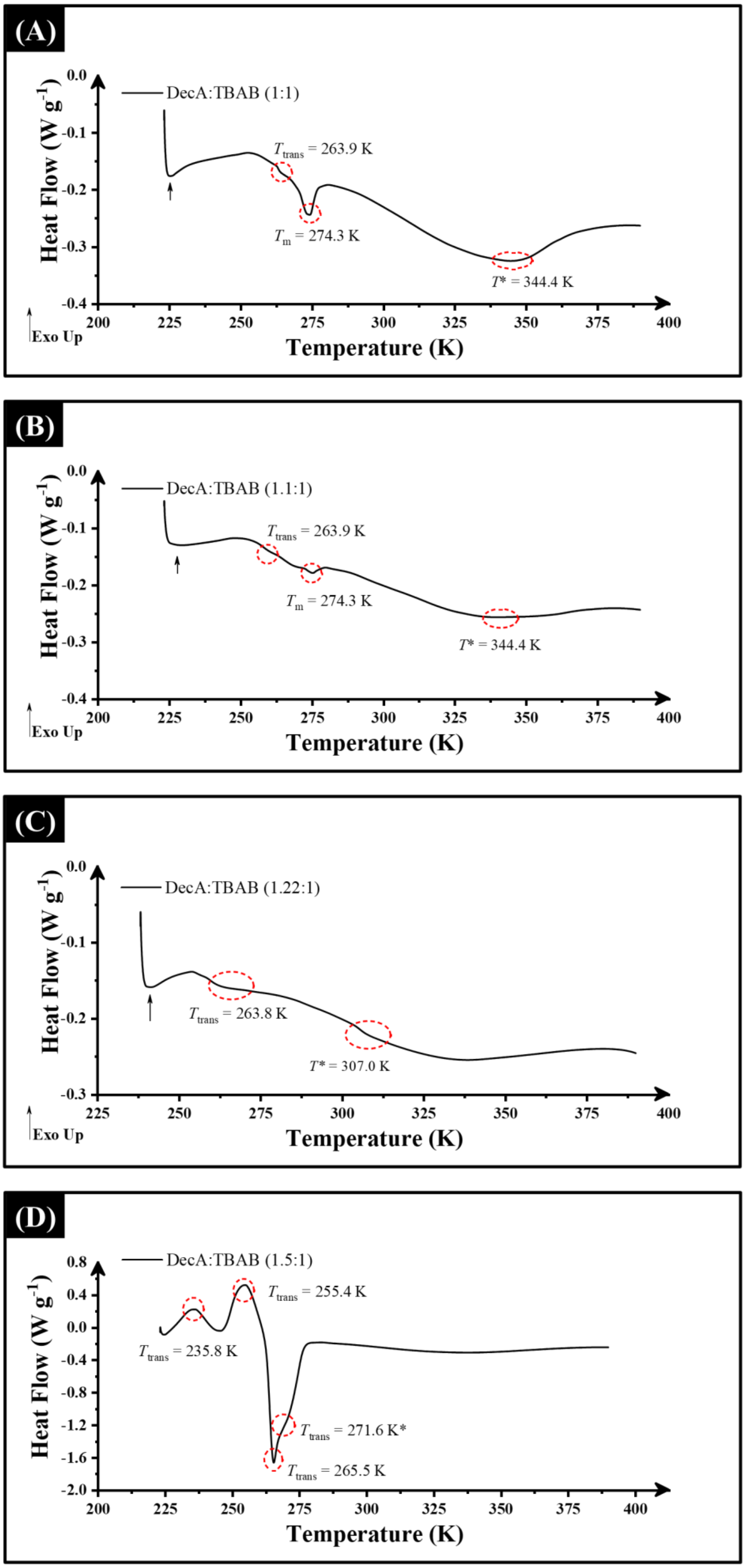
(A)
DecA:TBAB (1:1)
T trans = 263.9 K
T m = 274.3 K
T* = 344.4 K
Heat Flow (W g-1)
Temperature (K)
Exo Up
(B)
DecA:TBAB (1.1:1)
T trans = 263.9 K
T m = 274.3 K
T* = 344.4 K
Heat Flow (W g-1)
Temperature (K)
Exo Up
(C)
DecA:TBAB (1.22:1)
T trans = 263.8 K
T* = 307.0 K
Heat Flow (W g-1)
Temperature (K)
Exo Up
(D)
DecA:TBAB (1.5:1)
T trans = 255.4 K
T trans = 235.8 K
T trans = 271.6 K*
T trans = 265.5 K
Heat Flow (W g-1)
Temperature (K)

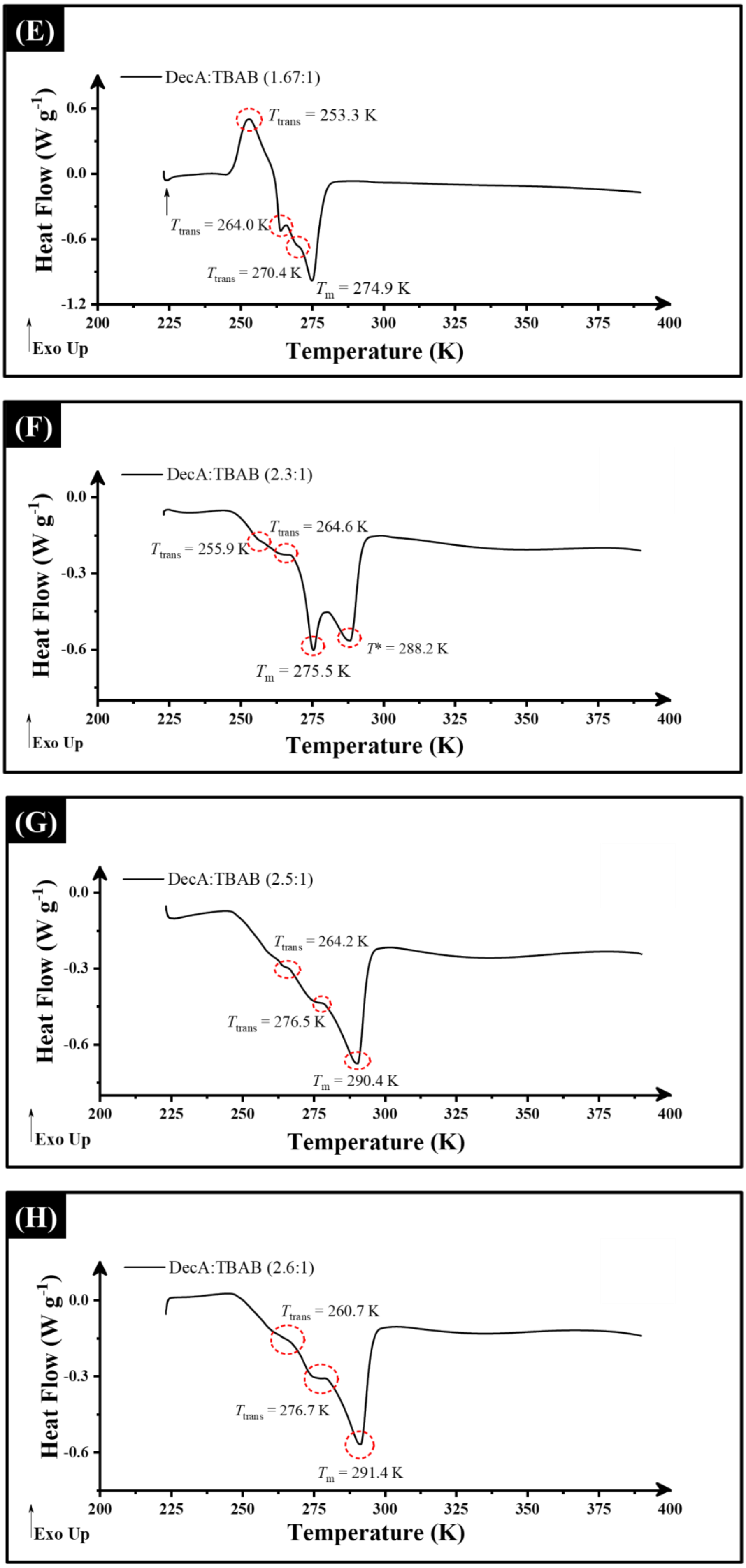
(E)
DecA:TBAB (1.67:1)
Heat Flow (W g-1)
0.6
0.0
-0.6
-1.2
T trans = 253.3 K
T trans = 264.0 K
T trans = 270.4 K
T m = 274.9 K
200
225
250
275
300
325
350
375
400
Exo Up
Temperature (K)
(F)
DecA:TBAB (2.3:1)
Heat Flow (W g-1)
0.0
-0.3
-0.6
T trans = 264.6 K
T trans = 255.9 K
T* = 288.2 K
T m = 275.5 K
200
225
250
275
300
325
350
375
400
Exo Up
Temperature (K)
(G)
DecA:TBAB (2.5:1)
Heat Flow (W g-1)
0.0
-0.3
-0.6
T trans = 264.2 K
T trans = 276.5 K
T m = 290.4 K
200
225
250
275
300
325
350
375
400
Exo Up
Temperature (K)
(H)
DecA:TBAB (2.6:1)
Heat Flow (W g-1)
0.0
-0.3
-0.6
T trans = 260.7 K
T trans = 276.7 K
T m = 291.4 K
200
225
250
275
300
325
350
375
400
Exo Up
Temperature (K)

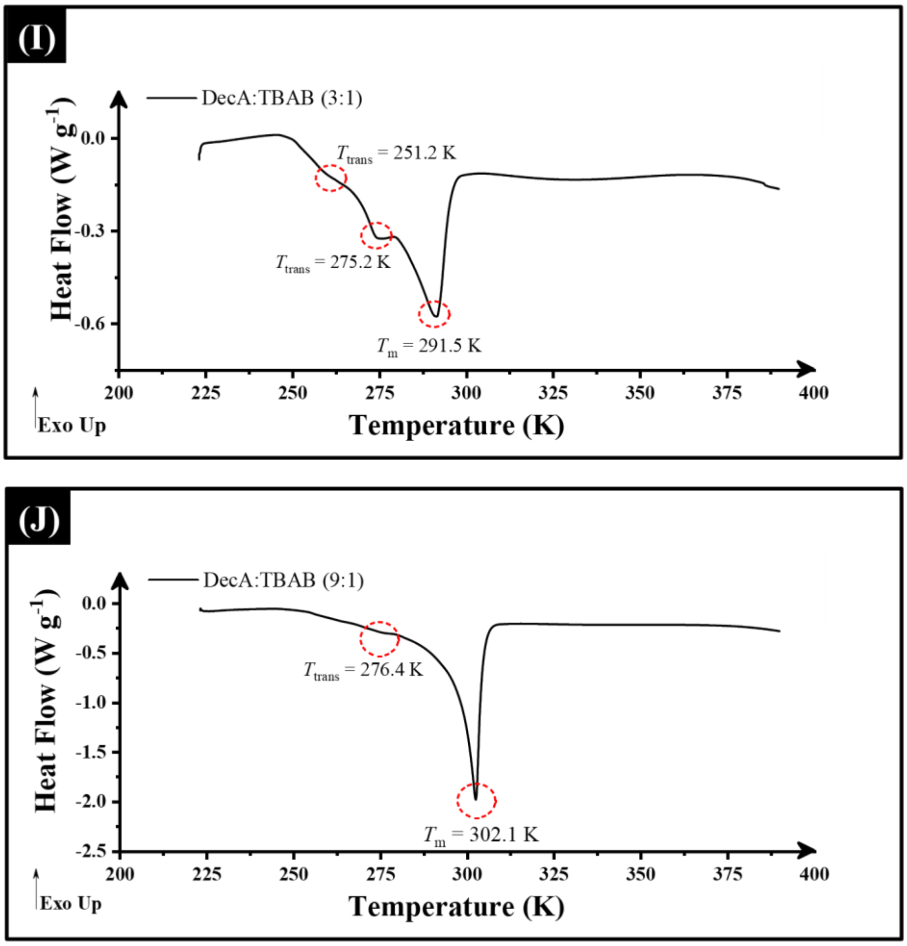

Figure S1. DSC curves with the thermal events for the different mixtures of DecA:TBAB. T_{trans}: temperature of phase transition; T_m: melting temperature; T*: temperature of the free fraction of the precursors (DecA or TBAB).

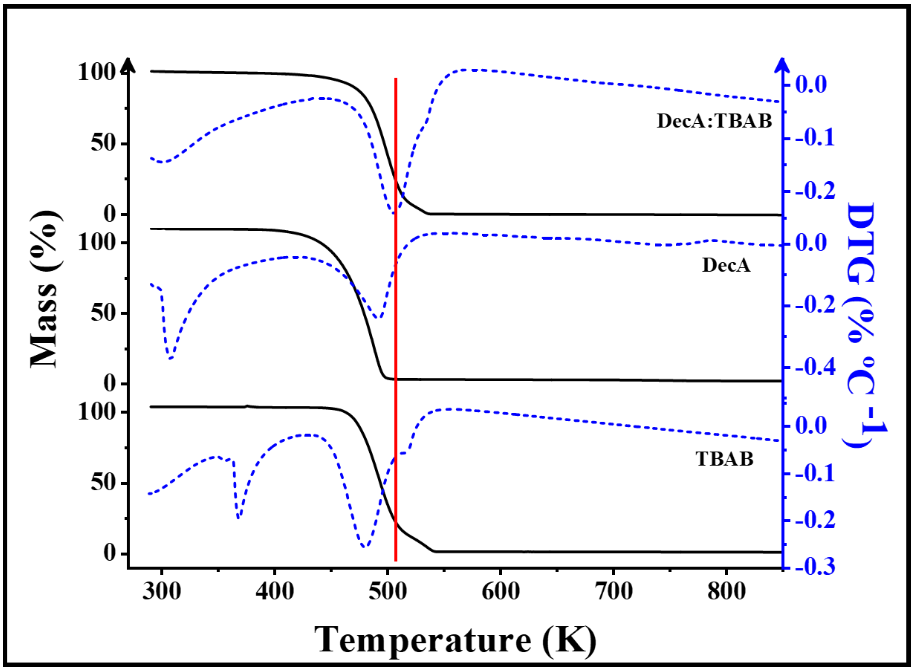

Figure S2. TG curves (solid lines) of HDES (DecA:TBAB, 1.85:1), decanoic acid (DecA) and tetrabutylammonium bromide (TBAB). DTG curves (dotted lines) of the HDES and its precursors (TBAB and DecA). Conditions: N_2 at 50 ml min^{-1}, sample mass of (10.0±0.2) mg in open α-alumina sample holders.

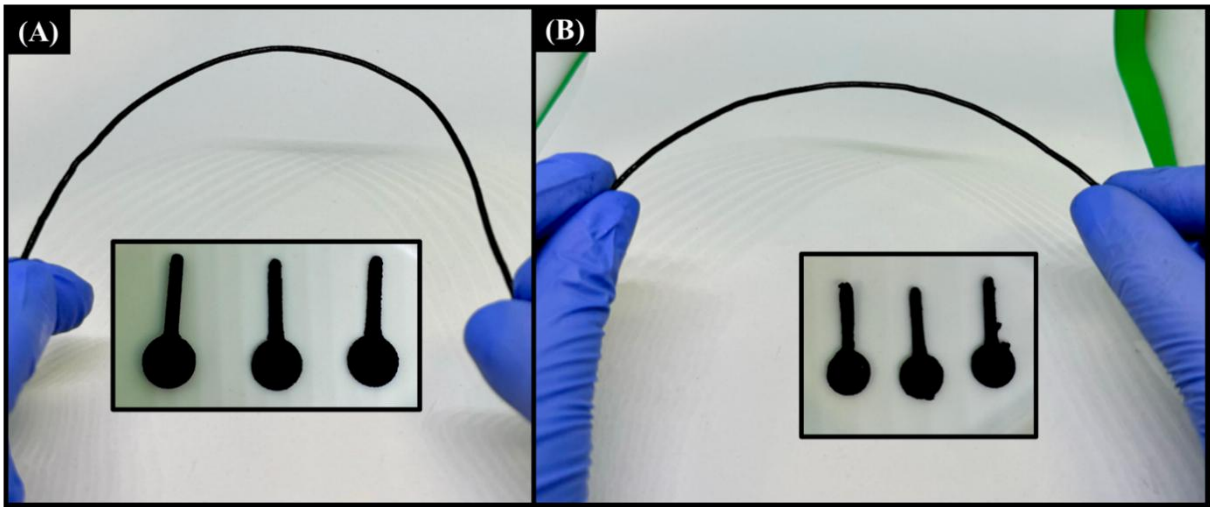

Figure S3. Images of the bespoke (A) CB/Cellulose/HDES/PLA and (B) CB/HDES/PLA filaments.

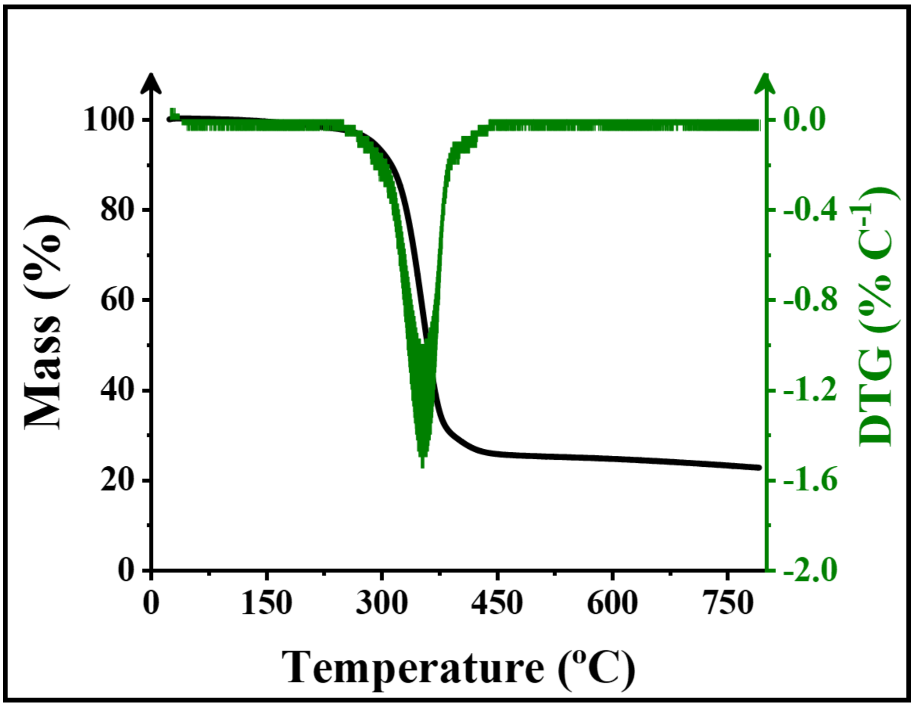

Figure S4. TG (black line) and DTG (green line) curves of the HDES-based filament. Conditions: N_2 at 100 mL min^{-1}.

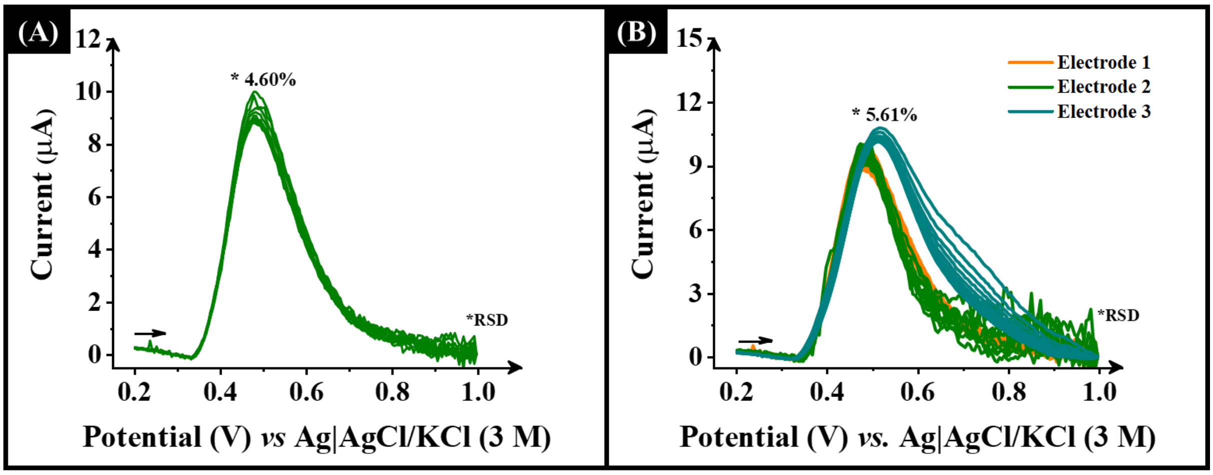

Figure S5. SW voltammograms obtained for repetitive measurements (N=10) in PBS pH 7.55 with 100 µM acetaminophen using (A) one electrode and (B) three different CB/Cellulose/HDES/rPLA electrodes as WE. SWV parameters: a = 20 mV, f = 25 Hz, ΔE = 5 mV.

Table S2. Comparison of the analytical parameters for the voltammetric determination of acetaminophen

Electrode	Technique	Linear range (μM)	LOD (μM)	Reference
CFμE	DPV	0.5 to 11	0.21	[1]
nano-Cu/GC_{EA}	SWV	4 to 28	0.35	[2]
CeBiO*x* NFs/SPE	DPV	2.5 to 130	0.2	[3]
MWCNT/CB filament	DPV	5 to 200	3.3	[4]
g-C_3N_4/SWCNTs/GCE	CV	20 to 200	0.76	[5]
$LaFe_{0.6}Ni_{0.4}O_3$/GCE	DPV	1 to 100	0.111	[6]
CB/Cellulose/HDES/PLA	SWV	5 to 300	0.12	This work

Key: CFμE: carbon fiber microelectrode; nano-Cu/GC_{EA}: copper nanoparticles modified electro-activated glassy-carbon electrode; CeBiO*x* NFs/SPE: CeBiOx nanofibers modified screen-printed electrode; MWCNT/CB filament; Multi-walled carbon nanotubes/carbon black filament; g-C_3N_4/SWCNTs/GCE: graphitic carbon nitride single walled carbon nanotube modified glassy carbon electrode; $LaFe_{0.6}Ni_{0.4}O_3$/GCE: glassy carbon electrode modified with $LaFe_{0.6}Ni_{(0.4)}O_3$ perovskite-type oxides.

References

[1] D. Bolaños-Méndez, J. Alvarez-Paguay, L. Fernández, P. F. Saavedra-Alulema, M. S. Veloz-Romero, P. J. Espinoza-Montero, *Chemosphere* **2024**, *346*, 140586.

[2] M. A. Kassem, M. I. Awad, M. Morad, B. A. Aljahdali, R. A. Pashameah, H. Alessa, G. I. Mohammed, A. Sayqal, *International Journal of Electrochemical Science* **2022**, *17*, 220441.

[3] F. Cao, Q. Dong, C. Li, J. Chen, X. Ma, Y. Huang, D. Song, C. Ji, Y. Lei, *Sensors and Actuators B: Chemical* **2018**, *256*, 143-150.

[4] R. D. Crapnell, I. V. S. Arantes, J. R. Camargo, E. Bernalte, M. J. Whittingham, B. C. Janegitz, T. R. L. C. Paixão, C. E. Banks, *Microchimica Acta* **2024**, *191*, 96.

[5] R. Subash, K. Madhivanan, R. Atchudan, S. Arya, A. K. Sundramoorthy, *Diamond and Related Materials* **2025**, *152*, 111940.

[6] L. Cao, H. Wang, B. Chen, S. Jiang, Z. Su, K. Chen, J. Wang, A. Xie, S. Luo, *Microchemical Journal* **2023**, *191*, 108851.
